# Mission *Tara* Microplastics: a holistic set of protocols and data resources for the field investigation of plastic pollution along the land-sea continuum in Europe

**DOI:** 10.1007/s11356-023-26883-9

**Published:** 2023-05-04

**Authors:** Jean-François Ghiglione, Valérie Barbe, Stéphane Bruzaud, Gaëtan Burgaud, Jérôme Cachot, Boris Eyheraguibel, Franck Lartaud, Wolfgang Ludwig, Anne-Leila Meistertzheim, Ika Paul-Pont, Stéphane Pesant, Alexandra ter Halle, Odon Thiebeauld, the Mission Tara Microplastics consortium, J. F. Ghiglione, L. Philip, C. Odobel, C. Pandin, M. Pujo-Pay, P. Conan, N. Luckas, V. Barbe, P. Wincker, S. Bruzaud, M. Kedzierski, M. Palazot, L. Soccalingame, G. Burgaud, A. Philippe, J. Cachot, B. Morin, E. Dusacre, C. Clérandeau, C. Lefebvre, B. Eyheraguibel, F. Lartaud, W. Ludwig, X. Durrieu de Madron, L. Weiss, A. L. Meistertzheim, I. Calves, K. Lebaron, E. Lavergne, I. Paul-Pont, A. Huvet, C. Dubreuil, S. Pesant, A. ter Halle, M. Albignac, O. Thiebeauld, K. Crenn, T. Gassane, L. Merakeb, C. Bauvois, F. Galgani, O. Gerigny, M. L. Pedrotti, G. Gorsky, F. Lombard, S. Alligant, C. Lacroix, L. Navarro, B. Sperandio, B. Diémé, C. Bowler, R. Troublé, R. Hentinger, A. Abreu, M. Thomas, M. Bourdreux, J. Schramm, C. Moulin, E. Bernollin, M. Hertau, S. Audrain, N. Bin, Y. Tournon, L. Boulon, F. Aurat, L. Blijdorp, C. Pire, S. Bin, C. Gicquel, M. Oriot

**Affiliations:** 1 CNRS, Sorbonne Université, Laboratoire d’Océanographie Microbienne (LOMIC)/UMR 7621, Observatoire Océanologique de Banyuls, Laboratoire d’Océanographie Microbienne, 1 Avenue Fabre, F-66650 Banyuls sur mer, France; 2 Research Federation for the Study of Global Ocean Systems Ecology and Evolution, R2022/Tara Oceans-GOSEE, Paris, France; 3 Génomique Métabolique, Genoscope, Institut François Jacob, CEA, CNRS, Univ Evry, Université Paris-Saclay, Evry, France; 4 UMR CNRS 6027, IRDL, Université Bretagne Sud, 56100 Lorient, France; 5 Univ Brest, INRAE, Laboratoire Universitaire de Biodiversité Et Écologie Microbienne, 29280 Plouzané, France; 6 Université Bordeaux, EPOC CNRS, EPHE, Université de Bordeaux, UMR 5805, 33600 Pessac, France; 7 CNRS, Université Clermont Auvergne, Institut de Chimie de Clermont-Ferrand (ICCF), UMR6296, Clermont-Ferrand, France; 8 CNRS, Sorbonne Université, Laboratoire d’Ecogéochimie des Environnements Benthiques (LECOB)/UMR 8222, Observatoire Océanologique de Banyuls, Banyuls Sur Mer, France; 9 CEFREM, UMR 5110, University of Perpignan - CNRS, 66860 Perpignan Cedex, France; 10 SAS Plastic@Sea, Observatoire Océanologique de Banyuls, Banyuls Sur Mer, France; 11 Ifremer, CNRS, IRD, LEMAR, Univ Brest, F‐29280 Plouzané, France; 12 European Molecular Biology Laboratory, European Bioinformatics Institute, Wellcome Genome Campus, Hinxton, Cambridge, CB10 1SD UK; 13 CNRS, Laboratoire des InteractionsMoléculaires EtRéactivité Chimique Et Photochimique (IMRCP), UMR 5623, Université de Toulouse, Toulouse, France; 14 ImmunRise Biocontrol France, Cestas, France

**Keywords:** Microplastics, Land-sea continuum, Toxicology, Plastisphere, Scientific expedition

## Abstract

The *Tara* Microplastics mission was conducted for 7 months to investigate plastic pollution along nine major rivers in Europe—Thames, Elbe, Rhine, Seine, Loire, Garonne, Ebro, Rhone, and Tiber. An extensive suite of sampling protocols was applied at four to five sites on each river along a salinity gradient from the sea and the outer estuary to downstream and upstream of the first heavily populated city. Biophysicochemical parameters including salinity, temperature, irradiance, particulate matter, large and small microplastics (MPs) concentration and composition, prokaryote and microeukaryote richness, and diversity on MPs and in the surrounding waters were routinely measured onboard the French research vessel *Tara* or from a semi-rigid boat in shallow waters. In addition, macroplastic and microplastic concentrations and composition were determined on river banks and beaches. Finally, cages containing either pristine pieces of plastics in the form of films or granules, and others containing mussels were immersed at each sampling site, 1 month prior to sampling in order to study the metabolic activity of the plastisphere by meta-OMICS and to run toxicity tests and pollutants analyses. Here, we fully described the holistic set of protocols designed for the Mission *Tara* Microplastics and promoted standard procedures to achieve its ambitious goals: (1) compare traits of plastic pollution among European rivers, (2) provide a baseline of the state of plastic pollution in the Anthropocene, (3) predict their evolution in the frame of the current European initiatives, (4) shed light on the toxicological effects of plastic on aquatic life, (5) model the transport of microplastics from land towards the sea, and (6) investigate the potential impact of pathogen or invasive species rafting on drifting plastics from the land to the sea through riverine systems.

## Introduction

Plastics are emerging pollutants and are now regarded as a key geological indicator of the Anthropocene because of their abundance and widespread distribution on Earth (Zalasiewicz et al. 2016). The mismanaged plastic waste (MPW) resulting from the production and inadequate disposal of waste on land is the major driver for plastic discharge to the ocean, with a potential annual transfer of 4.8 to 12.7 Mt per year (Jambeck et al. 2015). Plastic stocks at the ocean surface amount to tens to hundreds of kilotons (Eriksen et al. 2014), but this represents only a small percentage of the estimated annual discharge into the marine environment (Lebreton et al. 2017)—causing many to consider ‘the missing ocean plastic sink’. Several processes have been hypothesized to contribute to plastic sequestration in the ocean, such as sedimentation to the seabed, transport to the deep sea, stranding on beaches, fragmentation into undetectable particles, accumulation in living biomass, and biodegradation (Cressey 2016). However, only a few studies have questioned the amount of plastic carried to the ocean by rivers. An estimate of this plastic flux to the ocean has been established globally by several models (Cózar et al. 2014, Jambeck et al. 2015; Lebreton et al. 2017), but uncertainties are high because of the lack of data and complexities relating to the transport and fate of plastics from land to sea (van Calcar and van Emmerik 2019). A recent statistical re-analysis based on updated data on microplastic (MP) distributions demonstrated that previous estimates of river fluxes overestimated fluxes by up to three orders of magnitude (Weiss et al. 2021). Another compilation of raw data revealed that MPs were underestimated by up to one order of magnitude, with 24.4 trillion pieces in the world’s upper ocean (8.2 10^4^ ~ 57.8 10^4^ tons) (Isobe et al. 2021). While a strong link was shown between river discharge and plastic transport, the role of flood events is largely ignored by global plastic transport models albeit recent estimation alarms on these flood events to tenfold the global plastic mobilization potential (Roebroek et al. 2021). Analyzing plastic abundance by field observation data at the land-sea continuum is crucial to bridge the gap between the global riverine plastic emission extrapolated from models and actual observations (van Calcar and van Emmerik 2019). It will further help to provide a robust baseline for further estimation of the positive or neutral impacts of European directives or any initiative for the “plastic transition” aimed at reducing the quantity and composition of plastics reaching aquatic environments.

Toxic effects of plastics have been experimentally observed at the molecular, cellular, organ, individual, and population levels at various trophic levels (Anbumani and Kakkar 2018). Frequently reported effects were on feeding and digestive processes associated with altered energy and lipid metabolism that may lead to oxidative stress, inflammation, endocrine disruptor-like effects, abnormal development, and to some extent cell death (Kögel et al. 2020). Plastic additives such as the phthalates (PAEs), organophosphate esters (OPEs), and bisphenols (BPs) are furthermore likely to interact with marine living organisms through release from ingested plastic debris, assimilation by contact with the chemicals in the dissolved water phase, or by food web transfer (Fauvelle et al. 2021). Accumulation of waterborne toxic compounds on microplastics (MPs) (e.g., persistent organic pollutants and heavy metals) to concentrations higher than in the ambient water is another factor, probably linked to the large surface-volume ratio and hydrophobicity enabling MPs to accumulate waterborne toxic contaminants (e.g., persistent organic pollutants and heavy metals) (Holmes et al. 2012). The global transport of MPs has triggered concerns regarding the potential role that its mobility may represent towards influencing the long-range environmental transport of particle-bound chemicals. Beyond studying the effects of MPs exposure under controlled laboratory conditions, in situ experimental settings are now required to predict and evaluate the range of impacts plastic pollution may have, in interaction with other environmental stressors (Klasios et al. 2021).

The potential for long-range environmental transport of invasive species and microbial pathogens has also been evidenced. A large diversity of microbial hitchhikers living on plastics (the so-called “plastisphere”) have been described in various aquatic environments, including bacteria, single-cell algae, and fungi (Wright et al. 2020), raising important questions about the dynamic of this plastisphere and its role in plastic biodegradation, and whether plastics act as vectors for harmful pathogen species dispersal (Jacquin et al. 2019). Bacterial taxa considered as putative pathogens of fish (*Tenacibaculum* sp.) and invertebrates (*Vibrio splendidus*, *Phormidium* sp., and *Leptolyngbya* sp.) were sometimes more consistently and abundantly found on plastic compared to surrounding seawater (Dussud et al. 2018a). MPs colonized by pathogens may also pose threats to humans, as evidenced by the detection of human pathogenic bacteria on MPs collected along the Belgian (*Escherichia coli*, *Bacillus cereus*, and *Stenotrophomonas maltophilia*) and Brazilian (*Vibrio cholerae*, *V. vulnificus*, and *V. mimicus*) coasts (McCormick et al. 2014; Silva et al. 2019). Evidence is still missing to determine whether plastic debris could lead to the effective spread and prolonged persistence of pathogenic species across the land-sea continuum.

In the year 2000, the European Union launched an ambitious program called the Water Framework Directive (WFD) that requires a catchment management plan for all major European rivers to achieve “good ecological status” or “good ecological potential” by 2015 and 2027, respectively. The WFD is considered a cornerstone in sustainable freshwater and coastal ecosystem management, but it does not yet take into account MPs in the list of pollutants, probably because of the lack of available data in rivers. More data from the marine environment allowed the EU Marine Strategy Framework Directive (MSFD 2013) to include MPs as an aspect to be measured. There is an urgent need for more scientific field data of microplastic pollution in rivers to improve the European Water Framework Directive.

Mission *Tara* Microplastics was conducted for 7 months in 2019 to investigate the plastic pollution along nine major rivers in Europe, using an extensive suite of physical, chemical, and biological variables collected with consistent standardized methods at all sites. The main objectives of Mission *Tara* Microplastics are as follows: (1) draw a near-exhaustive census of the quantity and composition of MPs at the European land-sea continuum, (2) evaluate the proportion of MPs within the total debris stranded on river banks and beaches, (3) quantify the fluxes and predict the fate of MPs at the land-sea continuum, (4) investigate the level of divergence and standing variation of MPs toxicity on various organisms, (5) propose a new bioindicator of the presence and bio-accumulation of MPs based on mussel filter feeders, and (6) identify how the environment influences changes in the microorganism communities living on MPs (including pathogens) at the land-sea continuum. In this paper, we fully described the state-of-the-art procedures expended for the Mission *Tara* Microplastics to promote a holistic strategy for the immediate and long-term future study of plastic pollution in the land-sea continuum.

## A unique sampling strategy across Europe

**Fig. 1:**
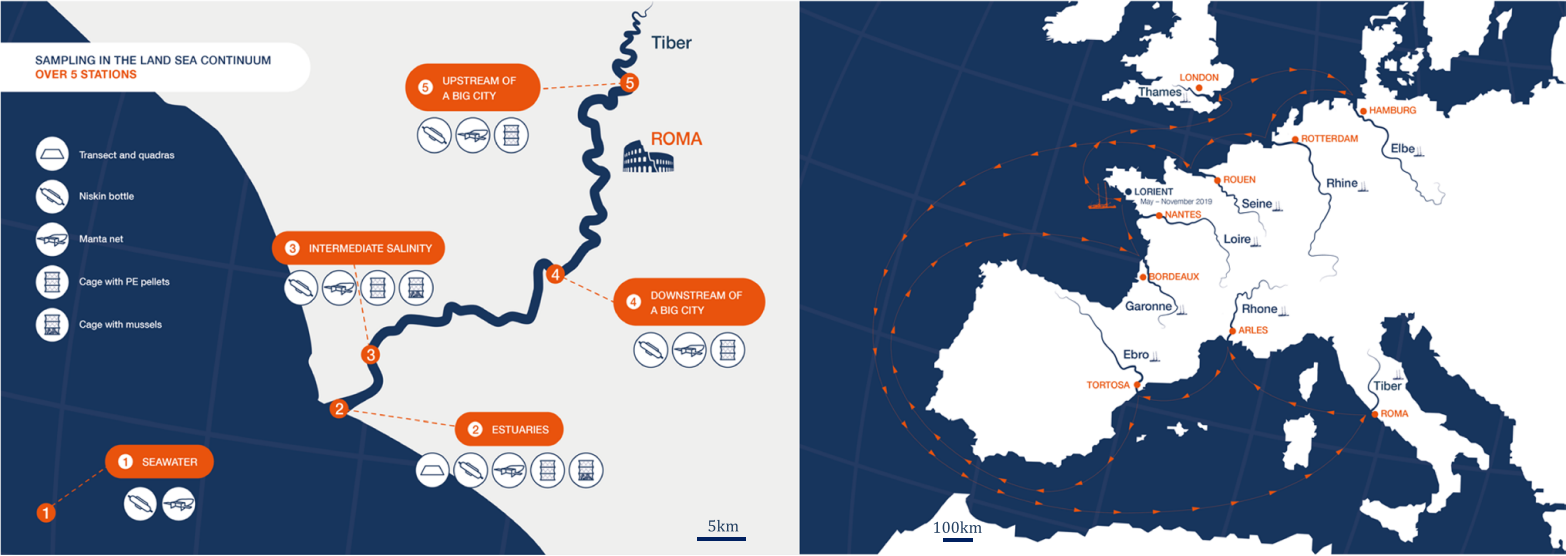
Sampling strategy in the land-sea continuum. Left: Example of sampling strategies in the Tiber River (Italy) along the salinity gradient from the sea (station 1), the outer estuary (station 2), intermediate salinity (station 3) to downstream (station 4), and upstream (station 5) of the first heavily populated city (Roma). Samples were taken on land (using transect and quadras), in the river (using Niskin bottle, Manta net, and cages with PE pellets or with mussels), and at sea (using Niskin bottle and Manta net only). Right: The route of the research vessel *Tara* for 7 months crossing 9 of the major rivers in Europe

Mission *Tara* Microplastics is a unique scientific expedition inspired by the importance of rivers as the most important channels of aquatic communication and commercial traffic in Western Europe. Harmonized sampling methodologies were used to provide an extensive suite of biophysicochemical parameters at 45 sampling sites along nine major rivers in Europe (Fig. 1). We chose four to five sampling sites along a salinity gradient from the sea and the outer estuary to downstream and upstream of the first heavily populated city located on each river, including London on the Thames (8.99 million habitants), Hamburg on the Elbe (1.82 million habitants), Rotterdam on the Rhine (0.62 million habitants), Rouen on the Seine (0.11 million habitants), Nantes on the Loire (0.30 million habitants), Bordeaux on the Garonne (0.25 million habitants), Tortosa on the Ebro (0.03 million habitants), Arles on the Rhone (0.05 million habitants), and Rome on the Tiber (2.87 million habitants). Water samples were routinely taken for 7 months onboard the French research vessel *Tara* or from a semi-rigid boat in shallow waters, whereas river banks and beaches were visited after landing scientists from the boat at the same location. The sampling was conducted along the approximately 9500 nautical miles-long track aboard *Tara*, with the participation of scientists succeeding in teams of five to eight persons. A team on land was dispatched one month before the arrival of *Tara* for site reconnaissance and for the deployment of cages containing pristine plastics or mussels.

## Sampling procedures, material, and methods

### Sampling, sorting, and chemical analysis of large and small microplastics in surface waters

#### Large microplastics (330 µm to 5 mm)

**Fig. 2:**
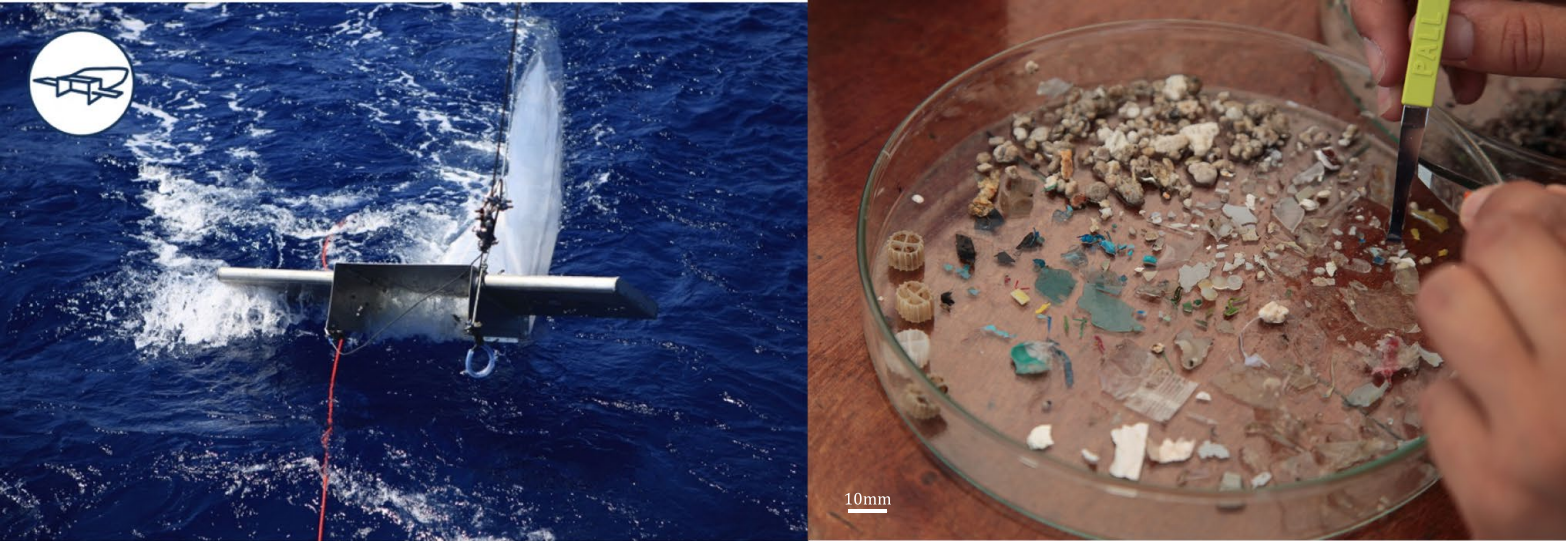
Photographs of the 330-µm mesh size manta trawl (left) and the following microplastic sorting with sterilized forceps (right) (*credit to Noélie Pansiot*, *Tara Ocean Foundation*)

Sampling for large microplastics was conducted using a 330-µm mesh size manta trawl (aperture of 30 × 80 cm, 2.5 m long nylon net, and 30 × 10 cm^2^ weighted cod end) (Fig. 2). Manta trawl was deployed at an approximate speed of 2.0 knots for 60 min in seawater and 10 min in rivers, in order to avoid clogging (especially in rivers). The volume of sampled water was measured using a mechanical flow meter located at the center of the net aperture (Hydo-bios, Kiel, Germany). After careful rinsing of the net with water from the sampling site, macro-debris of natural origin (algae, branches, leaves,…) were eliminated through rinsing above the collector. Particles with approximately 1 to 5 mm in size accumulated in the final volume of 1.0 l of the collector were transferred in glass Petri dishes, observed under a binocular magnifying glass, and sorted using alcohol/flame sterilized forceps (Fig. 2). The larger microplastic pieces (close to 5 mm) were cut into two parts in order to compare microbial counts and diversity on the same microplastic piece. The first, smaller part was fixed with 1% (v/v) glutaraldehyde for 30 min before freezing at − 20 °C until further electron microscopy analysis (protocol label **G330**, for glutaraldehyde and size of the net mesh). The second, larger part (or the entire when not cut) was immediately frozen in liquid nitrogen for the DNA extraction (protocol label **S330**, for sequencing—see “Mussels as bioindicators of small microplastics”) and further chemical identification by Fourier transform infrared spectroscopy FTIR (protocol label **S330_F**, see below). Pictures of each microplastic fragment were taken (Nikon D850 with a Micro-Nikkor 105 mm camera lens) to determine their dimensions, shapes, and colors (Fiji image processing software).

To analyze the entire quantity of microplastic collected by the 330-µm mesh size manta trawl, all remaining particles, which were not sorted by hand, were collected on 50-µm mesh size metallic filters, and frozen at − 20 °C before further analysis (protocol label **R330-F**). Once in the laboratory, the samples were thawed and subjected to an extraction process that removes organic (algae, branches, leaves, etc.) and inorganic matter (clay, sand, etc.). The samples were immersed in a 10% potassium hydroxide (KOH) solution (w/v) during 48 h at 40 °C and a second immersion in 30% hydrogen peroxide (H_2_O_2_) solution (v/v) was carried out during 12 h at room temperature if organic matter persisted after the filtration (Kedzierski et al. 2022). For very high concentrations of organic matter, a third step based on the Fenton’s reaction (Hurley and Nizzetto 2018) used H_2_O_2_ with an iron solution (ferrous sulfate heptahydrate FeSO_4_.7H_2_O) to catalyze the organic matter digestion reaction. Finally, the inorganic matter was removed by sodium iodide (NaI) density separation. After extraction, the particles larger than 500 □m were separated by sieving and disposed of with tweezers under a stereomicroscope (Motic SMZ-171) in a Petri dish. Then, particles were photographed (Nikon D850; Micro-Nikkor 105 mm camera lens) to determine their quantity, dimension, shape, and color (Fiji image processing software). In order to reduce the large number of particles to be processed by the time-consuming one-by-one FTIR analysis, a random subsampling using statistical analysis was applied, as previously described (Kedzierski et al. 2019, 2022).

An attenuated total reflection-Fourier transform infrared spectrometer (ATR-FTIR Vertex70v, Bruker, ATR Golden Gate) was used to determine the polymer composition and chemical characteristics of the sorted microplastics (both on board and in the laboratory) and FTIR spectra were identified using POSEIDON software (Kedzierski et al. 2019). For each of the polyethylene (PE) infrared spectra, the carbonyl index (CI), hydroxyl index (HI), and fouling index (FI) were determined, as previously described (Kedzierski et al. 2022).

We used a correction factor to evaluate the microplastic concentration (number of particles per m^3^) by using a wind-driven vertical mixing correction model taking into account individual particle properties (dimension, shape, and density) (Poulain et al. 2019). Finally, the microplastic concentration and the proportion of the different polymers were determined for each sample using R software (prop.test function which allows for testing the Equal Proportions; The R Core Team 2019).

#### Small microplastics (SMPs; 25 to 330 µm)

In order to collect the microplastic fraction down to 25 µm, another net was deployed on the side of the boat and at the same time as the 330-µm mesh size manta trawl deployed at the back of the boat. A separate net was used because the mesh size required to collect the SMP (25 µm) is distinct from the LMP (300 µm). Because LMP abundance is in the range of 0.1 to 1 particle.m^−3^, a total volume of several hundred of m^3^ of water is required to measure relevant concentration. SMP is much more abundant in number, then a smaller volume is needed with a mesh size of 25 µm. Based on our experience, several hundreds of liters are enough to collect SMP. Another important justification for this strategy is that net equipped with a 25-µm mesh size clogs very quickly, which rendered impossible its use to filtrate the several m^3^ required to monitor LMP concentrations.

The 25-µm nylon net (aperture of 32 × 82 cm with two floats placed on both sides for buoyancy, 2.5 m long net and 30 × 10 cm^2^ weighted cod end) was deployed for 10 min in seawater and 2 min in rivers at an approximately 2.0 knots speed. A mechanical flow meter located at the center of the aperture (Hydo-bios, Kiel, Germany) was used to determine the volume of sampled water. The net was thoroughly rinsed with on-site water and the collector was rapidly transferred to a calcinated 1-L glass jar equipped with a metallic cap in order to prevent any airborne contamination.

A new method was developed to analyze the small-microplastic fraction (25–330 µm) by pyrolysis coupled to gas chromatography and tandem mass spectrometry (Py-GC–MS/MS) (Albignac et al. 2022). The developments for microplastic analysis in Py-GC–MS are rather recent and less advanced compared to micro-FTIR spectroscopy (Primpke et al. 2020). One of the interesting aspects of the use of Py-GC–MS is that it does not have size limitations, and it is less time-consuming since all the microplastic pieces are pooled into one sample analyzed in one run (Okoffo et al. 2020). In the laboratory, the samples were first pre-filtered on a 500-µm inox grid and then filtered on a 25-µm inox grid. The dried suspended matter was homogenized by cryo-grinding (using the SPEX® SamplePrep 6775 Freezer/Mill cryogenic Grinder from Delta Labo, Avignon), followed by density separation using sodium iodide solutions (density of 1.56 g cm^−3^) to remove inorganic particles. The recovered matter was rinsed thoroughly on a 0.7-µm glass fiber filter (GF/F Whatman, from Sigma-Aldrich, St. Louis, MO, USA) and homogenized again by cryo-grinding. An aliquot of 2 mg of the dried sample was introduced and pyrolyzed at 600 °C. Details of the Py-GC–MS/MS development are given in an article recently published by a member of the *Tara* Microplastics consortium (Albignac et al. 2022). A total of six polymers among the most abundant polymers found in the environment were targeted, namely polyethylene (PE), poly(methyl methacrylate) (PMMA), polyethylene terephthalate (PET), polycarbonate (PC), polystyrene (PS), and polypropylene (PP).

A direct comparison between FTIR and Py-GC–MS analysis recently showed that the overall concentrations were in the same range with both techniques, with very few differences observed in polymer compositions (Primpke et al. 2020). Assumption of an extrapolated thickness from photographs of the two-dimensional geometric shape of each particle makes the conversion possible to convert between the number of particles and their mass (Simon et al. 2018; Poulain et al. 2019). Previous studies showed the efficiency of micro-FTIR for SMP analysis (Cowger et al. 2020), but others underlined the requirement of multiple-step preparation in the case of river samples (Adomat and Grischek 2021). The Py-GC–MS appears as a good strategy to analyze a large number of samples as generated in this project since it requires less sample purification steps with similar results obtained by both techniques (Primpke et al. 2020).

### Macro-, meso-, and microplastics on river banks and beaches

**Fig. 3:**
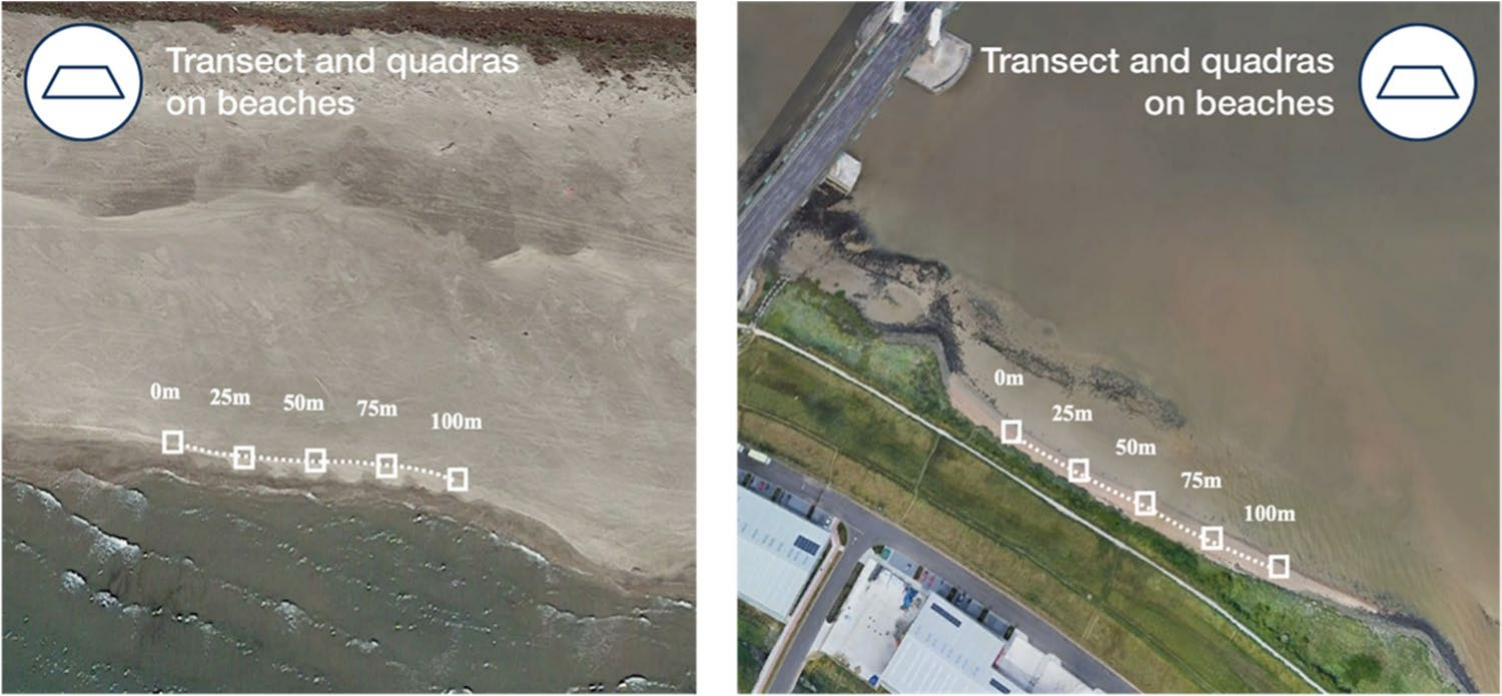
Sketch of the sampling protocol for macro-, meso-, and microplastics on beaches (left) and river bank (right). Squares are the five quadrats of 0.50 m length sides (0.25 m.^2^) along the high tide (dotted line)

The sampling protocol was based on the Marine Strategy Framework Directive guidance (MSFD 2013) together with recommendations by the Cedre (center of documentation, research, and experimentation on accidental water pollution) (Lefebvre et al. 2021). All visible plastics deposited onto the shoreline and riverbanks were collected 1 m above and below a 100-m longitudinal transect following a line formed by the last high tide (Fig. 3). They were separated in three categories: microplastic 0.5 to 5 mm, mesoplastic 0.5 to 2.5 cm, and macroplastic > 2.5 cm (protocol label HPL-Micro, HPL-Meso, and HPL-Macro). Along the 100-m longitudinal transect, quadrat of 0.50-m length sides (0.25 m^2^) were laid on the tide line at 0 m, 25 m, 50 m, 75 m, and 100 m. GPS coordinates were taken at the beginning and the end of the transect (0 and 100 m). Meticulous inspections were conducted by checking the quadrat surface, firstly without disturbance and secondly by checking each organic or mineral item constituting the tide line and removing them to check also if plastic-like items were not hidden by other materials. All suspected plastics (e.g., colored items, hardly breakable, no ornamentation, no squeaking under stainless steel pliers) that were visible with the naked eye were recovered and placed in 250-mL clean aluminum trays to avoid plastic contamination (protocol label *HPQ*). Each tray was closed and annotated with the date of sampling, the name of the river, operator, the position of the quadrat (0, 25, 50, 75, 100 m), and the geographic coordinates of the transect. All samples were preserved on board *Tara* in the dark at − 20 °C for further analysis.

Visual sorting was performed as recommended by GESAMP (Kershaw et al. 2019). Microplastics were counted, and, using a stereomicroscope (LEICA MZ7.5), size (maximal length and width), shape (i.e., pellet, fragment, film, fiber/filament or foam, microbead), color (white, blue, black, yellow, green), opacity (transparent or opaque), and surface particle roughness (smooth or rough) were recorded for each sampled particle. Items were weighed for each shape category of each quadrat (Mettler Toledo AE240S; scale division: 0.01 mg) and then summed up to obtain the overall weight of the particles from each quadrat. The chemical characterization of MPs was performed using FTIR, as described above. All statistical comparisons were performed using R Studio (version 1.3.1093). When data were normally distributed, variable comparisons were made using ANOVA tests and post hoc tests (Student–Newman–Keuls (SNK)). Due to the very small number of samples and the high heterogeneity of the MPs, most data were not normally distributed and then the Kruskal–Wallis non-parametric test was carried out if needed by Mann–Whitney tests (wilcox.test function with paired = TRUE argument on R software) to identify differences between sites.

### Mussels as bioindicators of small microplastics

Bivalves are filter feeders, which means they filter the surrounding water through their gills to feed on phytoplankton and other particles. Mussels have a large filtration capacity, ranging from 1 to 25 µm, at rates of 20 to 25 L of water/day for the *Mytilus* spp. (Boromthanarat and Deslous-Paoli 1988). In addition to their food, these organisms also accumulate pollutants, sometimes at higher concentrations than are found in their surrounding environment, which has led to their use in various monitoring programs such as Mussel Watch in the USA, MYTILOS in the Mediterranean Sea, and RINBIO/ROCCH in France (Beliaeff et al. 2002). Due to low selectivity when filtering, mussels ingest large quantities of MPs (mainly between 1 and 25 □m), e.g., 4 MPs.individual^−1^ measured in commercial mussels from China (Li et al. 2015) and 0.2 g of MPs.g^−1^ for mussels in Europe (Van Cauwenberghe et al. 2015), which led the scientific community to consider mussels as a potential bioindicator of MPs (Li et al. 2019). This assumption assumes that mussels maintain their filtering capacity when exposed to plastics allowing the bioaccumulation of MPs related to environmental concentrations. A recent paper demonstrated the possible use of bivalves as efficient bioindicators of microplastic pollution in Korean coasts (Cho et al. 2021).

Mission *Tara* Microplastics offered the first in situ approach to investigate the use of bivalves as efficient bioindicators of microplastic pollution in Europe, based on catch and recovery experiments from contrasted habitats of the main European estuarine waters. Depending on the area of the nine European rivers targeted and their geographic distribution, *Mytilus edulis* or *Mytilus galloprovincialis* (and likely hybrids for the Atlantic coastline) were sampled from close sites, stained with calcein to provide a chronological mark into the shell, placed into cages (*n* = 30), previously stained with calcein to provide a chronological mark into the shell (3 stations including seawater, estuarine, and intermediate salinity stations on the nine rivers). A survey of the health status of organisms was provided through a combination of integrated physiological parameters, including the stress response at the molecular level, energy reserves, shell growth rate, and reproductive status. Microplastics filtered/bioaccumulated by mussels were measured after digestion of the organic tissues with KOH and novel techniques based on Py-GC–MS to characterize both the size and the type of micro- and nanoplastics (Yakovenko et al. 2020). The shell growth rate was assessed through observation of the linear extension from the calcein mark recognized by fluorescence microscopy (Andrisoa et al. 2019). Energy reserves were measured from lipid, protein, and carbohydrate contents from colorimetric assays (see the Method in Chapron et al. 2021), and the molecular stress response was inferred through the assessment of chaperones levels in the whole body by the enzyme-linked immunoabsorbent assay (ELISA) (Meistertzheim et al. 2009). Such combinations of integrated physiological parameters ensure a survey of the health status of organisms, which is crucial to ensure that mussels maintain their filtration rate whatever the environmental settings.

### Water sampling and metadata

#### Temperature and salinity

A thermosalinograph (TSG, Seabird SBE45) was installed onboard the RV *Tara* for surface temperature and conductivity measurements at a sampling frequency of 0.1 Hz. Discrete vertical measurements from 0 to 30-m depth were also done at each sampling station using a portable Sontek CastAway CTD probe (Conductivity, Temperature, Depth) (ADCPro, France) attached to the rope holding the 8-L Niskin bottle. The analytical precision was 0.01 °C for temperature and 0.01 to 0.05 for salinity.

#### Water sampling

**Fig. 4:**
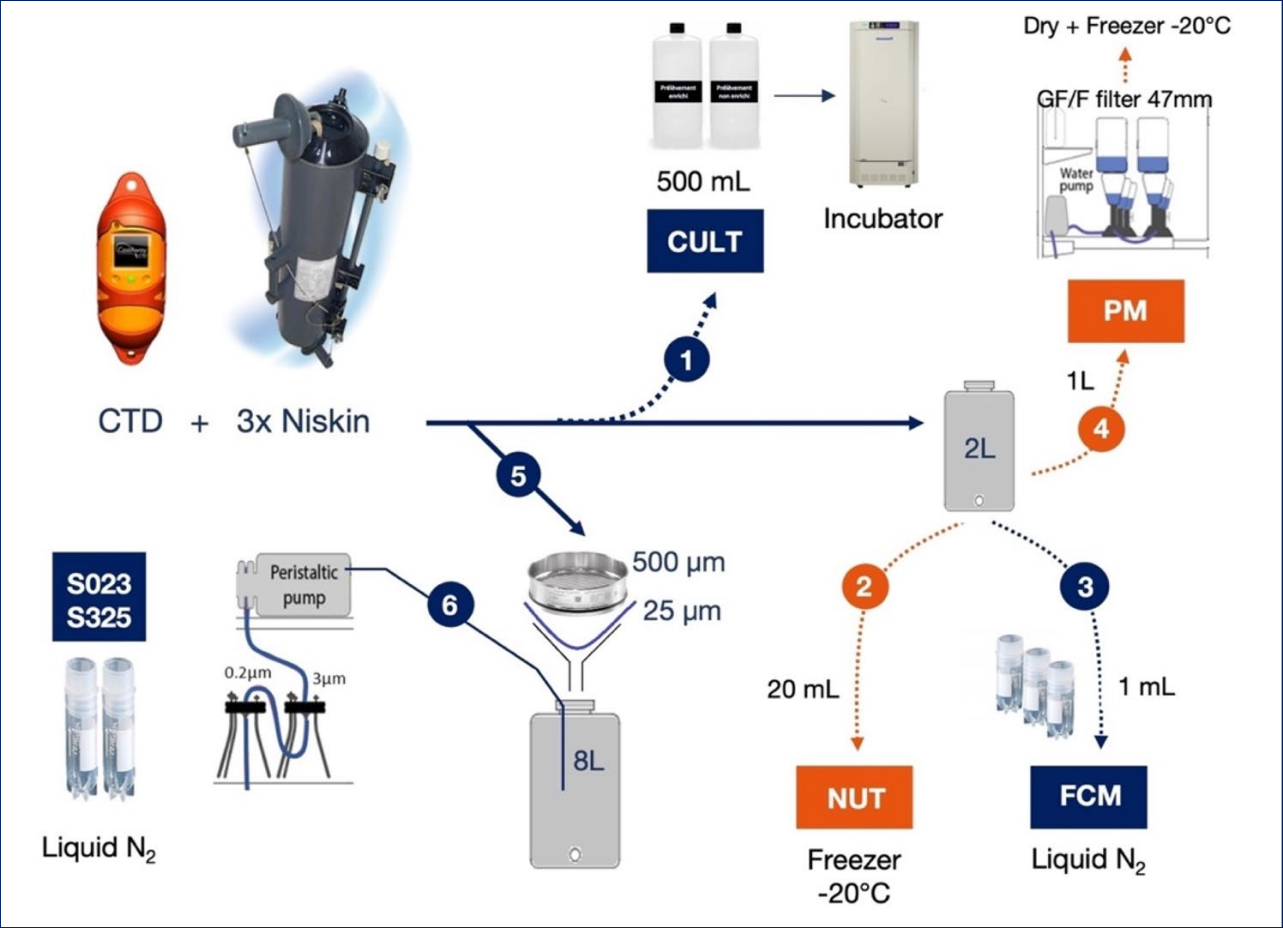
Schematic diagram of the water sampling system, showing the setup of the various parameters analyzed from the same sampling station. CULT, microbial culture; NUT, nutrients; FCM, flow cytometry; PM, particulate matter; S023, sequencing on DNA extracted from 0.2-µm filters after 3-µm prefiltration; S325, sequencing on DNA extracted from 3-µm filters after 25-µm prefiltration

At each water sampling station and just before the concomitant 330-µm manta trawl and 25-µm net deployments, three 8-L Niskin bottles were triggered below the surface and pooled in an acid-cleaned tank. Water subsamples were then transferred to a set of specific devices for nutrients, particulate matter, microbial counts, and diversity analysis (Fig. 4).

#### Nutrients, chlorophyll a, and particulate matter

An 18-mL subsample of water was filtered through a glass syringe fitted with a Whatman AnoDisc-Paradisc 0.45-µm filter and placed in a 20-mL polyethylene scintillation vial (protocol label NUT, Fig. 4). Another 8 mL were placed in a 20-mL polyethylene scintillation vial for ammonium (NH_4_^+^) analysis. The samples were frozen at − 20 °C and stored until analysis. Upon return to the laboratory, nitrate (NO_3_^−^), nitrite (NO_2_^−^), phosphate (PO_4_^3−^), and dissolved silica (Si(OH)_4_^−^) concentrations were measured on a continuous flow Seal-Bran luebbe® AutoAnalyzer III, whereas NH_4_^+^ determinations were performed by fluorimetry on a Jasco FP-2020 fluorimeter (Holmes et al. 1999). The analytical precision of NO_3_^−^, NO_2_^−^, PO_4_^3−^, and Si(OH)_4_^−^ is ± 0.02 µM, ± 0.01 µM, 0.02 µM, and ± 0.05 µM, respectively, and ± 5 nM for NH_4_^+^.

Water subsamples (from 100 to 500 mL) were filtered on pre-combusted glass fiber filters (Whatman GF/F, 25 mm, 450 °C, 12 h) and then frozen in liquid nitrogen for chlorophyll or dried at 60 °C and stored in a desiccator for particulate matter until further analysis (protocol label PM, Fig. 4). Suspended particulate matter (SPM) concentrations were determined by differences between the dry weights of the respective filters before and after filtration. Particulate organic matter (POM) was measured by dry combustion on a CHN 2400 Perkin Elmer analyzer (detection limit: 0.1 mg of C) after decarbonatization through subsequent rinsing of the filters with phosphoric (1 M) and hydrochloric (2 M) acid. Finally, chlorophyll *a* was estimated according to Yentsch and Menzel (1963) with an analytical precision of 0.03 mg L-1 for chlorophyll *a*.

### Estimation of plastic fluxes at the land-sea continuum in Europe

Evaluation of the general MP levels is important since previous studies proposed much lower levels in European compared to Asian and/or African rivers (Lebreton et al. 2017; Schmidt et al. 2017). However, MP concentrations in rivers can be highly variable at seasonal and spatial scales (Weiss et al. 2021 and references therein). The nine rivers sampled during the Mission *Tara* Microplastics were characterized by a large variability of total suspended sediment (TSS) and particulate organic carbon (POC) contents (data not shown), indicating that together they cover contrasted hydrological conditions. They should consequently allow at least an order of magnitude estimate of the average MP levels in European rivers, as well as give insights into the prevalent relationships between MP and other particulate materials in rivers. Moreover, since sampling was conducted along salinity gradients from pure freshwater to fully marine conditions, the collected data may also give insights into the question of whether estuaries may act as filters, for example, through sequestration of MPs from surface layers through biological productivity and/or modification of hydrodynamic conditions and density gradients. We are of course aware that, since our sampling strategy corresponds to “one shot” sampling which does not take into account, for example, temporal variability in relation to tidal hydrodynamics, our data may not allow full responses to these questions. However, if non-conservative mixing along the salinity gradients could be detected systematically, this may nevertheless initiate further research based on more exhaustive temporal sampling strategies.

General riverine plastic loads should be established in terms of mass fluxes as this allows direct comparison with the floating plastic masses detected at the ocean surfaces. However, in most cases, plastic in rivers is only detected in the form of individual debris (items per m^3^ or per L) and appropriate algorithms for converting debris to mass fluxes are hence the bottleneck in the determination of realistic mass concentrations (Weiss et al. 2021). Conversely, the selection of the appropriate filtering method is important since this determines the size classes of the retained MPs. For evaluating the approximate MP concentrations in the sampled rivers, we only focussed on the MPs which were collected by the 330-µm mesh size manta trawl (see “Large microplastics (330 μm to 5 mm)”). The weight of the MP debris could not be measured individually, but all were photographed in order to determine their dimensions and shapes in combination with Fiji image processing software tools. This allows the determination of their approximate 2D surface areas which then can be, based on standard geometries and data training from other rivers and aquatic environments (Constant et al. 2020; de Carvalho et al. 2021; Kedzierski et al. 2022), converted into average volumes and average MP weights.

Riverine load estimates are derived by multiplying the average MP mass concentrations with the corresponding daily discharge values of the sampled rivers. The latter will be collected from the most downstream gauging stations from hydrological monitoring networks which are generally freely accessible on the Internet. For all rivers, a meta-database will be established from publicly available databases on the general drainage basin characteristics which could be potential drivers for MP exportation from river basins. Among them, population density and associated socioeconomic parameters are of particular interest. All the data will finally be processed by standard statistical methods and tools in order to examine major differences between rivers, freshwater, and marine environments and to develop the most appropriate statistical models for large-scale budget calculations.

### Microbial life on plastics and surrounding waters at the land-sea continuum

#### Microbial richness and diversity

**Fig. 5:**
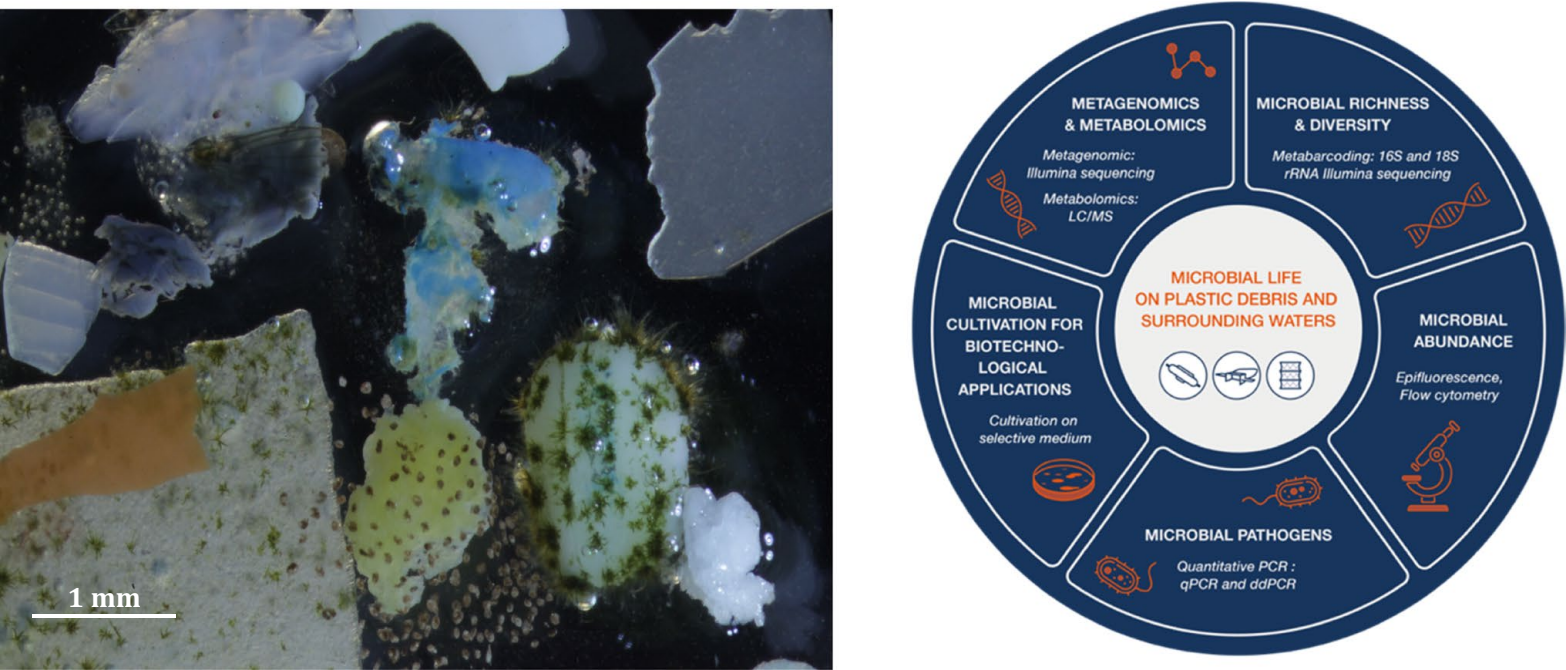
Illustration of microbial life on plastics (left; *credit to Christian Sardet*) and complementary set of studies performed during the Mission *Tara* Microplastics to investigate the microbial life on plastics and in the surrounding waters (right, *related methodologies are indicated in italic*)

Plastics provide novel habitats for the microbial communities occurring on their surfaces and represent a unique ecological niche which appears distinct from surrounding seawater and other natural and/or non-natural particles, such as wood, glass, ceramic…, both in terms of prokaryotic and microeukaryotic diversity (Jacquin et al. 2019). Mission *Tara* Microplastics sampling strategy and the associated metabarcoding workflow will allow (i) to delve deeper into the concept of niche partitioning and confirm on a European scale whether plastics select for specific microbial communities, (ii) to assess whether environmental parameters (with a focus on salinity gradients) have a significant influence on the community composition compared to the type of plastic (type of polymers), and (iii) to determine whether overlapping and non-overlapping microbial groups are made of dominant or rare taxa. The originality of our approach is to generate an exhaustive amount of data for bacteria, archaea, and microeukaryotes (with a specific focus on fungi), using plastic and surrounding seawater samples collected along a salinity gradient on nine rivers on a European scale (Fig. 5).

Microplastic samples (**called S330** for Sequencing MPs sorted from the 330-µm manta net), as well as the surrounding organic particle-attached (**called S3-25** for sequencing 3-µm filters after sequential pre-filtration on 25-µm filters) and free-living fractions (**called S02-3** for sequencing 0.2-µm filters after sequential pre-filtration on 3-µm filters) (see Fig. 4) were thawed before DNA extraction by classical phenol–chloroform method, as previously described (Dussud et al. 2018b). After cellular lysis and before the phenol–chloroform extraction, all MPs were characterized by FTIR to determine their polymer composition (see above, “Sampling, sorting, and chemical analysis of large and small microplastics in surface waters”) to better understand whether plastic types trigger specific communities, as recently emphasized (Pinto et al. 2019) or overturned (Kettner et al. 2019). DNA was quantified by spectrophotometry (GeneQuant II, Pharmacia Biotech).

Amplification for metabarcoding (MetaB) was performed on the same DNA extracts using a nested-PCR approach for (i) the bacterial/archaeal 16S V3–V5 region using 515F-Y and 926R primers (Parada et al. 2016) after a preliminary amplification using 27 F and 1492R primers (Miller et al. 2013), (ii) the eukaryotic 18S V9 region using the 1389F and 1510R (Amaral-Zettler et al. 2009) after a preliminary amplification using the universal eukaryotic primers Euk-A and Euk-B (Koid et al. 2012), and (iii) the fungal hypervariable internal transcribed spacer region 2 (ITS2) using 5.8S-Fun and ITS4-Fun primers (Taylor et al. 2016) after a preliminary amplification using ITS1F and ITS4 primers (Rämä et al. 2016). Sequencing is performed on Illumina NovaSeq for 16S V3-V5 and 18S V9 regions and on MiSeq for ITS2 region by Genoscope (Evry, France).

One of the unique aspects of the Mission *Tara* Microplastics is to homogenize the protocols used for metabarcoding data processing for Bacteria, Archaea, Eukaryotes, and Fungi from the same sequencing approach and using a unique metabarcoding pipeline named SAMBA (v3.0.1), which is a fairly scalable workflow to conduct reproducible metabarcoding analyses. This will generate findable, accessible, interoperable, and reusable (FAIR) scientific data. Our idea is to process uniform up-to-date bioinformatic processes (Qiime 2 data cleaning (Bolyen et al. 2018), DADA2 ASV inference (Callahan et al. 2016), dbOTU3 for ASV clustering (Olesen et al. 2017), MicroDecon for sample decontamination (McKnight et al. 2019), ASV taxonomic assignation and phylogenetic reconstruction, and ANCOM differential abundance testing (Mandal et al. 2015), and statistical analyses allowing alpha- (diversity indices and taxonomic diversity) and beta-diversity analyses to evaluate the variation of microbial communities in a single sample and between samples, respectively. The workflow also allows for wider comparison through the integration of datasets from other scientific studies as a meta-analysis approach in order to confirm the ecological trends and distribution patterns that were highlighted when studies were analyzed independently.

#### Metagenomic and metabolomic approaches to explore functional diversity

##### Metagenomic analysis (MetaG)

Metagenomic analysis of microbial populations occurring on marine plastics allows for a higher resolution, providing another stratum of information about microbial community structure, as well as accessing the genetic potential of the plastisphere. The gene content makes it possible to define metabolic networks of the plastispheres by highlighting the pathways that are present. Databases such as COG and PFAM corresponding to orthologous genes and to protein families respectively are standard tools to explore the content of metagenomic genes. Comparative analysis of MetaG sequences (plastics, free-living, and organic particle-attached fractions) makes it possible to focus on the main predictive functions of genes. Gene pools are highlighted using shotgun sequencing and provide a detailed prediction of the biodegradation potential in situ but also the variety of processes managed by the plastisphere such as adaptation to the environment, pathogenicity, cell-to-cell interactions, secretion system, chemotaxis, xenobiotic compound degradation, and others.

Contrasted results on whether or not the microorganisms occurring on plastics are really degrading them have already been obtained, from speculation (Bhagwat et al. 2021) to clear evidence from gene pools which can reveal enrichment of enzymes engaged in polymer degradation such as esterases, depolymerases, hydrolases, laccases,… (Pinnell and Turner 2019; Purohit et al. 2020). These first hints definitely call for further research studies to delve deeper into the plastisphere genetic potential using a metagenomic-based approach. In this frame, the variety of samples collected during the Mission *Tara* Microplastics will allow us to better understand microbial assemblages along with their lifestyles and biogeochemical activities, and their patterns that depend on biotic and abiotic factors (Fig. 5). Based on metagenomic sequencing data, metagenome-assembled genomes (MAG) will be reconstructed leading to a landscape of the most representative organisms of the communities compared to the rare biosphere (Tully et al. 2018). The exploration and comparative genomic analyses of the MAGs may answer, amongst other things, questions about plastic biodegradation.

Because of low DNA concentrations after extraction, a total of five DNA microplastic samples (S330) from the same sampling site were pooled. In parallel, around 10 g of the encaged polyethylene pellets incubated for 1 month in cages on site were placed in 50 mL tubes and immediately frozen in liquid nitrogen to avoid changes in metabolite levels and to limit residual enzymatic activity (protocol label **CS-PE** for Sequencing of encaged PE pellets). Illumina sequencing was performed on NovaSeq (2 × 150 nt) to reach around 100 million reads per sample. The bioinformatics analysis of the metagenomic data was done using the SqueezeMeta pipeline (Tamames and Puente-Sánchez 2019). It includes co-assembly procedures with read mapping and co-assembly of a large number of metagenomes and internal checks during the assembly and binning steps (see for examples in Odobel et al. 2021).

##### Metabolomics

Environmental metabolomics was employed to study the biochemical interactions of microbial communities living on plastics and aimed at describing the metabolic status of the plastisphere in natural environments (Lankadurai et al. 2013). The approach aims to extract, directly from the whole microbial community, as many naturally occurring metabolites as possible. The detection and quantification of the metabolites can reveal upregulated and downregulated biomarkers that are characteristic of the biological processes occurring in the biofilm (Jones et al. 2013; Macel et al. 2010). This metabolic profiling is used as a snapshot of the response of the plastisphere to the overall environmental stressors across rivers and streams. It can help to identify the processes that control the fate of plastic, such as biodegradation or toxicity.

Metabolic profiling was used in comparative approaches to assess if metabolic patterns associated with biological processes are preserved among the various sampled environmental conditions. The variety of sampling sites allowed us to assess the impact of biotic conditions such as microbial diversity, and abiotic conditions such as salinity, temperature, organic matter content, and metabolic activity of the plastisphere. A comparison of inter- and intra-river variability was performed to assess and contrast metabolic activities between samples, rivers, and associated sampling sites (Fig. 5).

For metabolomic analysis, 1.5 g of the 1-month engaged polyethylene pellets were weighed out in 15 mL tubes and extracted with 4 mL of a solvent mixture of water/methanol/acetonitrile [1:2:2] solution kept in an ice bath, thoroughly vortexed for 5 min, and incubated 1 h at − 20 °C. The supernatant of 4 mL of solvent was transferred to a new tube and centrifuge (10 min, 4 °C, 12,000 g) to remove cell debris. The procedure was repeated once and the two supernatants were pooled. The final extracts (8 mL) were evaporated at 10 °C for approximately 48 h using a refrigerated centrivap concentrator equipped with a centrivap cold trap at − 50 °C (LabConco; ThermoScientific). The dry extracts were resuspended in 200 µl of 50/50 acetonitrile/water, and an aliquot of 50 μL was dedicated to LC–MS analyses. Moreover, for five aliquots of 10 μL of each sample, 10µL was collected and was mixed together for serving as quality controls (QC) through LC–MS analyses. QC samples were analyzed at the beginning and after every 8 samples, analyzed randomly, in order to assess the reproducibility of the measurements.

Metabolomics liquid chromatography coupled to mass spectrometry analyses was performed with an UHPLC Ultimate 3000 system (Thermo FisherScientific) coupled to a TimsTOFExactive Orbitrap mass spectrometer (BrukerThermo Fisher Scientific) equipped with an electrospray (ESI) source and operating in the positive and negative ion modes. The chromatographic separations are performed using a Kinetex EVO C18 Acquity HSST3 column (2.1 × 1500 mm; 1.87 µm-WatersPhenomenex) operating at 30 °C and an injection volume of 5 µl. The flow rate is fixed at 0.245 mL/min with 0.1% of formic acid in water (A) and 0.1% of formic acid in acetonitrile (B) for mobile phases at the following gradient: initial, 0–2 min, 100% A; 2–15 min linear gradient to 0% A; 15–22 min, 0% A; 22–22.10 min linear gradient to 100% A; following by 4 min of washing and reconditioning of the column equilibration wash with 100% A. 95% A; 0–7.5 min linear, 1% A linear; 7.5–8.5 min, 1% A; 8.5–9 min, 95% A linear, 9–11 min 95% A following by washing and reconditioning of the column. The mass spectrometer is operated in positive and negative ion modes and the detection was performed with a full scan from m/z 50 to 1000 in both ionization modes with capillary voltage at 3.2 kV and − 3 kV, respectively, and a capillary temperature set at 320 °C. The detection is performed with a full scan from m/z 80 to 1200 in both ionization modes using a resolution set at 70,000 at m/z 200. Xcalibur Software (version 4.1) is used for data acquisition and analysis.

MS raw data were processed using the web-based platform Galaxy platform framework (Giacomoni et al. 2015). XCMS software implemented in the Workflow4Metabolomics4Workflow platform is used to process raw data for feature detection, alignment, and grouping (Smith et al. 2006). The data matrices from LC–MS (positive and negative ionization mode) are subjected to multivariate analyses in order to highlight discriminant features between samples from different sites (partial least square discriminant analysis, PLS-DA). Identification of fragmentation spectra is completed by interrogation of public databases like (KEGG (Kanehisa and Goto 2000; Kanehisa et al. 2016), MetaCyc (Caspi et al. 2014), HMDB (Wishart et al. 2013), and MET-LIN (Smith et al. 2005)).

#### Transfer of pathogens at the land-sea continuum

While putative pathogenic bacteria have been regularly detected on MPs floating at sea, raising concern about disease spread and emergence over large geographical scales, the occurrence of other harmful microorganisms such as viruses on MPs remains largely unknown (Moresco et al. 2022). The vast majority of studies addressing the identification of plastisphere-associated species rely on bioinformatics analyses of high-throughput sequencing data of the gene encoding the small subunit of ribosomal RNA (16S & 18S rDNA; metabarcoding). Although this approach provides a robust evaluation of the plastisphere richness and diversity, it does not provide sufficient phylogenetic resolution for reliable identification of pathogenic species and their virulence factors (Thompson et al. 2009). The specificity of the approach performed in the Mission *Tara* Microplastics project is to couple high-throughput sequencing analyses to access community structure with PCR (qPCR and ddPCR) analyses targeting specific pathogenic species such as viruses (e.g., *ostreid herpesvirus)* and bacteria (e.g., *Vibrio crassostreae*, *V. splendidus*) responsible of disease outbreaks affecting bivalve mollusk species along the European coastlines. These pathogens were first selected based on (i) their previous detection of plastic debris and/or their ability to sorb on polymers (Vincent-Hubert et al. 2021); (ii) their abundance in continental, estuarine, and coastal environments (Vincent-Hubert et al. 2021; Paul-Pont et al. 2014); and (iii) their relevance for animal health (Le Roux et al. 2016). PCR assays will be performed using CFX Connect for qPCR (Bio-Rad, California, USA) or QX200 Droplet Digital PCR for ddPCR (Bio-Rad, California, USA). All reactions were run in triplicate together with negative (no template) and positive (pure culture) controls. A standard curve used a total of 6 dilutions ranging from 1 × 10^1^ to 1 × 10^6^ copies/5 μL.

PCR reactions will be performed on replicate DNA extracts of the encaged polyethylene pellets (protocol label CS-PE samples) all along the nine European rivers in order to identify potential sources of pathogens/microplastics that to our knowledge, were never studied before (Fig. 5). These samples with known immersion time and life history are to be compared with floating PE microplastics collected with the 330 μm Manta net (i.e., protocol label S330) from the same sites whenever possible (i.e., when DNA concentrations were sufficient). Being transitional ecosystems, estuaries concentrate on strong environmental gradients (e.g., salinity and suspended matter) that may influence the persistence and the virulence of pathogens associated with the plastisphere. For example, the salinity gradient represents one of the most important factors preventing marine–freshwater cross-colonization (Logares et al. 2009).

#### Microbial counts

Large microplastic pieces fixed on 1% (v/v) glutaraldehyde were used for microbial counting by epifluorescence microscopy observations using an Olympus AX70 PROVIS after 4’, 6-diamidino-2-phenylindole (DAPI) staining (Odobel et al. 2021) (Fig. 4). Cell counts were also determined using a FACSCanto II flow cytometer (BD Bioscience, San Jose, CA) equipped with a blue laser (488 nm, air-cooled, 20 mW solid-state) after nucleic acid dye SYBR Green I staining (Mével et al. 2008). Flow cytometry was directly used for cell counts in waters or after cell detachment pre-treatment (pyrophosphate followed by a sonication step) on large microplastic debris (Dussud et al. 2018a). Cell counts are expressed as the number of cells.mm^−2^ for polymers and as the number of cells.L^−1^ for seawater.

#### Microbial cultures for the screening of new molecules for biotechnology

The large biodiversity of marine microorganisms produces a wide and unexplored repertoire of bioactive compounds with original structures and enzymatic activities, which are sometimes not found across terrestrial organisms (Bhakuni and Rawat 2005). As an example, a recent study unveiled 40,000 new biosynthetic gene clusters from natural and phylogenetically diverse marine microbial communities, some of which belong to previously undescribed phylogenetic groups (Paoli et al. 2022). In addition, there is emerging evidence indicating that marine microorganism-derived molecules can exhibit potent antimicrobial and anti-infective activities (Suleria et al. 2016; Mayer et al. 2020). For example, turbinmicin was recently retrieved from a sea squirt microbiome as having a potent efficacy towards multiple-drug-resistant fungal human pathogens (Zhang et al. 2020). Plastics were shown as a new niche for a large diversity of microorganisms, which differ from the free-living or organic particle-attached fractions living in the surrounding seawater (Dussud et al. 2018b). Here, we aimed to explore the plastisphere as a potential for a wide chemical diversity to identify antimicrobial and anti-infective compounds that may be further exploited as novel biocontrol and therapeutic agents (Fig. 5).

Water samples were obtained directly from the water surface using an 8-L Niskin bottle or through the 330-µm mesh size manta trawl by collecting the 1.0 l of the collector (filtrated water). To allow microalgae survival, water samples were diluted or not in L1RS30 media (Guillard and Hargraves 1993) and 500 µL of each undiluted and diluted sample were distributed in a 48-well plate before incubation (18 °C with day/night: 14 h/10 h). Direct isolation of microalgae was also performed by micro-pipetting under the microscope. Bacteria were directly grown on Marine Agar plates and incubated at 18 °C before shipping. Microalgae clones were maintained in culture in ventilated 50-mL flask, whereas bacterial clones were obtained by successive isolation on marine agar and stored at − 80 °C as glycerol stocks. Using these methodologies, we were able to isolate and maintain 254 microalgae strains and 522 bacterial strains. In order to find lead compounds for agronomic (agricultural) and therapeutic (pharmaceutical) applications, the cultivated microorganisms were screened for their ability to inhibit the growth of plant or human pathogens (biocide activity) or to enhance plant or human immune pathways (elicitor/immune-modulator activity).

### Plastic toxicity and pollutants attached to plastics

In the last decade, growing evidence of the impact of plastic on organisms has been described, with three types of stress: (i) mechanical stress from the accumulation of plastics into organisms, (ii) chemical stress from additives included in the plastic or pollutants adsorbed on their surface, and (iii) biological stress from microorganisms, including pathogens (Hantoro et al. 2019). To our knowledge, the toxic effects of MPs have never been studied on a salinity gradient close to estuaries.

#### Lixiviation of additives and pollutants from plastics

Plastics consist of a polymer matrix to which additives are added to modify the physicochemical properties of the plastic (dyes or plasticizers) or to increase its resistance to UV, heat, fire, etc. These chemicals are not bound to the polymer matrix by covalent bonds and can, during the aging of the plastic, diffuse inside and outside the plastic. More than 10,000 chemical substances are used in plastic production, 2400 of which are of special concern as persistent, bioaccumulative, or toxic (Wiesinger et al 2021). The aging of plastic during its use or after its release into the natural environment under the action of UV radiation, heat, mechanical stress, among others, or even during its ingestion under the action of acidic gut pH and mechanical and enzymatic attacks, leads to an accelerated release of additives but also of micro and nanoplastics and oxidation products. Being hydrophobic compounds with high reactivity, plastics can also adsorb chemical contaminants released into the aquatic environment, including toxic persistent organic pollutants (POPs: PAHs, PCBs, etc.) and heavy metals (Fe, Mn, Al, Pb, etc.) (Rios et al. 2007; Holmes et al. 2012).

Our extraction protocol has been adapted from Pannetier et al. (2019). Two grams of MPs (1 month in situ encaged polyethylene pellets or MPs sampled on 0.25 m^2^ quadrats in river banks and beaches) were weighted and added to 4 mL of dimethyl sulfoxide (DMSO, Sigma) into an amber glass flask of 8 mL. After 16 h of shaking at 150 rpm at 20 °C (KS501 orbital shaker, IKA Werke), the liquid phase was immediately separated from the solid one with a glass Pasteur pipette and stored at − 20 °C in an amber flask. Three replications of extraction were performed for each sample. A blank of extraction with virgin pellets was prepared in the same conditions as for samples.

The chemical composition of the extracts was analyzed by GC/MS. Two microliters of the aliquot of DMSO extract were dried under a nitrogen flux and were concentrated in 200 µL of hexane. Analyses were performed using an Agilent 6890N GC coupled to an Agilent 5973 Mass detector, and chromatographic separation was obtained on a J@W Scientific HP-5MS (30 m/0.25 mm/0.25 µm) column. Mass spectrometer acquisitions were made in scan mode, from 50 to 800 m/z with 70 eV of electronic impact ionization energy. Compound identification was performed by comparison with an in-house compound database and with the NIST 1.7 standard reference database.

#### Toxicity tests

Ingestion of MPs and the diffusion of pollutants and additives in the digestive tract and their internalization can subsequently exert a chemical stress on organisms when ingested, as shown on the clam *Tegillarca granosa* (Tang et al. 2020) on the Pacific oyster, *Crassostrea gigas* (Bringer et al. 2020, Tallec et al. 2020), or different species of teleost fish (Cormier et al. 2021). The co-presence of MPs and pollutants raises the question about a potential synergistic effect aggravating the toxic effects of each isolated contaminant as observed on scallops (Xia et al. 2020).

Effects of waterborne contaminants were investigated here by measuring the toxicity carried out of polyethylene pellets incubated for one month in the different estuaries and also from plastic and microplastic sampled on beaches.

##### Microtox® assay

The Microtox® is a standardized acute toxicity assay using the marine bacteria *Vibrio fisheri* (ISO 11348 2007). Bioluminescence inhibition was measured on serial 1:2 dilutions of extracts starting from the highest concentration at 1%. Each assay was run in triplicate. Bioluminescence was measured after 30 min of exposure and compared with negative (NaCl 2% + 1% DMSO) and positive controls (200 mg/L potassium dichromate) using a Microtox 500 analyzer (Azur environmental).

##### Oyster embryo-larval assay

The acute embryotoxicity assay with *Crassostrea gigas* embryos was performed according to the standardized protocol NF ISO 17244 (2015). MPs extracts were tested at the final concentration of 0.1% in triplicates on freshly fertilized embryos. Embryos were incubated in a climatic chamber for 24 h at 24 °C in the dark. At the end of the exposure, developmental arrests and abnormal D larvae were recorded according to Bringer et al. (2020). Embryotoxicity of extracts was compared to a control with 0.1% of DMSO.

### Fair data and metadata resources

Mission *Tara* Microplastics project adopted the early deposition of metadata and data using infrastructure services hosted and operated by the European Bioinformatics Institute (EMBL-EBI). These include the BioSamples registry, the European Nucleotides Archive (ENA), and the MGnify bioinformatics pipeline, which seamlessly share content with services offered by the NCBI and BBNJ as part of the International Nucleotide Sequences Data Centre (INSDC).

Data provenance, including basic geo-references (i.e., date, time, latitude, longitude, and depth) and methodology (e.g., sampling devices, sample processing time, filtration volume, storage conditions) were captured throughout the expedition using unique sample identifiers (barcode stickers) affixed on the sample container and on pre-defined metadata logsheets. All logsheets were digitized and archived, and metadata were extracted semi-automatically and validated manually. The environmental context of all samples is documented using a combination of field measurements (i.e., temperature, salinity, nutrients, particulate matter elemental composition, and concentration) and controlled fields from the GSC MIxS community (https://www.ebi.ac.uk/ena/browser/view/ERC000024), the environmental ontology ENVO (https://www.ebi.ac.uk/ols/ontologies/envo), the Marine Regions gazetteer (https://www.marineregions.org/), and proprietary fields that further improve FAIRness.

## Conclusions

The widespread distribution of plastic debris from macro- to nano-scales undoubtedly threatens nearly all known ecosystems and their associated living macro and microorganisms communities, thus further perturbing ecosystem functioning and biogeochemical cycling. Multi-disciplinary approaches are needed to address research and societal questions to fill knowledge gaps in the current understanding of environmental plastic fluxes from the land to the sea through riverine systems and distribution, as well as the impact on biotic compartments and environmental health. The underway sampling protocols and analytical strategies presented here offer a first step toward these goals and the coming data will be invaluable for catapulting the science on plastics into the modern ‘big data’ era. Rich cross-disciplinary data as well as environmental metadata will be generated for each sample, in a structure that is consistent and interoperable with other expeditions. This is the case, for example, for the current *Tara* Microbiomes mission (2020–2022), that sampled in South America (Amazon) and several African (Congo, Gambia, Senegal) rivers with the same protocols. We anticipate that the unprecedented scale of these projects with consistent protocols will complement the growing knowledge about plastic pollution around the world, a quest for which advances are urgently needed with regard to the current European and international initiatives.

## Data Availability

The datasets and materials used and/or analyzed during the current study are available on reasonable request.
